# Exosomes of human placenta-derived mesenchymal stem cells stimulate angiogenesis

**DOI:** 10.1186/s13287-017-0660-9

**Published:** 2017-10-03

**Authors:** Motohiro Komaki, Yuri Numata, Chikako Morioka, Izumi Honda, Masayuki Tooi, Naoki Yokoyama, Hirohito Ayame, Kengo Iwasaki, Atsuko Taki, Noriko Oshima, Ikuo Morita

**Affiliations:** 1 Department of Nanomedicine (DNP), Graduate School of Medical and Dental Science, Tokyo Medical and Dental University, 1-5-45, Yushima, Bunkyo-ku, 113-8510 Tokyo, Japan; 2 Department of Pediatrics and Developmental Biology, Graduate School of Medical and Dental Science, Tokyo Medical and Dental University, 1-5-45, Yushima, Bunkyo-ku, 113-8510 Tokyo, Japan; 3 Department of Comprehensive Reproductive Medicine, Graduate School of Medical and Dental Science, Tokyo Medical and Dental University, 1-5-45, Yushima, Bunkyo-ku, 113-8510 Tokyo, Japan; 4 Department of Periodontology, Graduate School of Medical and Dental Science, Tokyo Medical and Dental University, 1-5-45, Yushima, Bunkyo-ku, 113-8510 Tokyo, Japan; 5 Life Science Department, Research and Development Division for Applied Technology, Research and Development Center, Dai Nippon Printing Co., Ltd, 250-1, Wakashiba, Kashiwa-city, Chiba 277-0871 Japan; 6 Current Address: Kanagawa Dental University, Yokohama Clinic, Tsuruya-cho 3-31-6, Kanagawa-ku, Yokohama, Kanagawa, 221-0835 Japan

**Keywords:** Placenta, Mesenchymal stem cells, Angiogenesis, Conditioned medium, Exosomes

## Abstract

**Background:**

The therapeutic potential of mesenchymal stem cells (MSCs) may be attributed partly to humoral factors such as growth factors, cytokines, and chemokines. Human term placental tissue-derived MSCs (PlaMSCs), or conditioned medium left over from cultures of these cells, have been reported to enhance angiogenesis. Recently, the exosome, which can transport a diverse suite of macromolecules, has gained attention as a novel intercellular communication tool. However, the potential role of the exosome in PlaMSC therapeutic action is not well understood. The purpose of this study was to evaluate PlaMSC-derived exosome angiogenesis promotion in vitro and in vivo.

**Methods:**

MSCs were isolated from human term placental tissue by enzymatic digestion. Conditioned medium was collected after 48-h incubation in serum-free medium (PlaMSC-CM). Angiogenic factors present in PlaMSC-CM were screened by a growth factor array. Exosomes were prepared by ultracentrifugation of PlaMSC-CM, and confirmed by transmission electron microscopy, dynamic light scattering, and western blot analyses. The proangiogenic activity of PlaMSC-derived exosomes (PlaMSC-exo) was assessed using an endothelial tube formation assay, a cell migration assay, and reverse transcription-PCR analysis. The in-vivo angiogenic activity of PlaMSC-exo was evaluated using a murine auricle ischemic injury model.

**Results:**

PlaMSC-CM contained both angiogenic and angiostatic factors, which enhanced endothelial tube formation. PlaMSC-exo were incorporated into endothelial cells; these exosomes stimulated both endothelial tube formation and migration, and enhanced angiogenesis-related gene expression. Laser Doppler blood flow analysis showed that PlaMSC-exo infusion also enhanced angiogenesis in an in-vivo murine auricle ischemic injury model.

**Conclusions:**

PlaMSC-exo enhanced angiogenesis in vitro and in vivo, suggesting that exosomes play a role in the proangiogenic activity of PlaMSCs. PlaMSC-exo may be a novel therapeutic approach for treating ischemic diseases.

**Electronic supplementary material:**

The online version of this article (doi:10.1186/s13287-017-0660-9) contains supplementary material, which is available to authorized users.

## Background

Mesenchymal stem cells (MSCs) are tissue-derived cells with self-renewing ability and can differentiate into various cell lineages. Various studies have reported previously that MSCs could elicit therapeutic effects via differentiation and/or secretion of factors such as growth factors, cytokines, and chemokines [1]. Furthermore, MSCs contribute to the repair of tissues damaged by ischemic diseases, including stroke, myocardial infarction, and cerebral infarction [2–4]. However, the mechanisms are not fully understood.

The placenta is a transient organ that maintains fetal tolerance and constitutes a rich reservoir of MSCs [5, 6]. Since MSCs are readily isolated from the placenta without invasive procedures, their use does not elicit ethical concerns [7, 8]. Previously, several studies have demonstrated that term placenta-derived MSCs (PlaMSCs) enhanced angiogenesis. For example, Kong et al. [9] reported that injection of human PlaMSCs enhanced microvessel formation in the skin wounds of diabetic rats, and these cells secreted proangiogenic molecules including vascular endothelial growth factor (VEGF), hepatocyte growth factor (HGF), basic fibroblast growth factor (bFGF), transforming growth factor beta (TGF-β), and insulin-like growth factor-1 (IGF-1). Furthermore, König et al. [10] reported that paracrine effects of conditioned medium (CM) from human PlaMSCs enhanced endothelial cell viability, migration, and tube formation, and elevated the secretion of proangiogenic proteins such as angiogenin, angiopoietin-1, angiopoietin-2, GRO, interleukin (IL)-6, IL-8, monocyte chemoattractant protein 1 (MCP-1), thrombopoietin, Tie2, and VEGF.

Recent studies including ours have reported that MSCs secreted extracellular vesicles, including exosomes [11–14], which are membrane nanovesicles released from various types of cells after fusion of multivesicular bodies (MVBs) with the plasma membrane. Exosomes contain various molecules including proteins, mRNA, and microRNA (miR), and have received increased attention as novel intercellular communication tools [13, 15]. Nevertheless, the function of exosomes is not fully understood. In the current study, we examined the role of exosomes in the angiogenic activity of PlaMSC-conditioned medium (PlaMSC-CM).

## Methods

### Cell culture and preparation of conditioned medium

Human term placentas were obtained from individuals who underwent elective cesarean section at 38 weeks of gestation. All participants were healthy Japanese women aged 31–37 years. Patients with a history of infection (including that by human immunodeficiency virus, hepatitis B virus, hepatitis C virus, or syphilis), underlying diseases (diabetes, hypertension, or regular use of medication), or obstetric complications (pregnancy-induced hypertension, threatened premature delivery, placenta praevia, or gestational diabetes) were excluded from this study. The study protocol was approved by the Ethics Committee for Clinical Research at the Tokyo Medical and Dental University (#1102). All study participants provided written informed consent.

PlaMSCs were isolated from the chorionic plate and villous chorion of term placentas (*n* = 8) following previously described methods with some modifications [14, 16]. The phenotype of the PlaMSCs was characterized by flow cytometric analysis of cell surface antigens, including tests for cluster of differentiation (CD)11b, CD31, CD34, CD44, CD45, CD73, CD90, and CD105. PlaMSCs were detached from culture dishes using 0.05% Trypsin/0.53 mM EDTA (Wako Pure Chemical Industries, Ltd, Tokyo, Japan), washed, and added to polystyrene tubes with a filter top (BD Bioscience, Heidelberg, Germany). The cells were incubated with either antigen-specific antibodies or isotype controls for 15 min on ice. Excess antibodies were removed by washing the cells with phosphate-buffered saline (PBS). Flow cytometric analyses were conducted on the BD FACSAria cytometer (BD Bioscience), using BD FACSDiva software.

To evaluate the differentiation potential of PlaMSCs, osteogenic, adipogenic, or chondrogenic differentiation was induced using osteogenic, adipogenic, or chondrogenic differentiation media (hMSC Differentiation BulletKit; Lonza, Walkersville, MD, USA), respectively, according to the manufacturer’s instructions. Human umbilical vein endothelial cells (HUVECs) were purchased from Lonza, and cultured on collagen-coated dishes (Iwaki, Shizuoka, Japan) in Endothelial Cell Growth Medium 2 (EGM2; Lonza). Human bone marrow-derived MSCs (BMMSCs) were purchased from Lonza, and cultured in MSC growth medium MSCGM (Lonza). CM was prepared using methods described previously [14]. Briefly, PlaMSCs were cultured in MSCGM, and the medium was changed to serum-free Dulbecco’s modified Eagle’s medium (D-MEM; Thermo Fisher Scientific, Waltham, MA, USA) when the cells reached ~ 80% confluence. The CM was collected after 48 h of incubation.

### Recovery and characterization of exosomes

Exosomes were recovered from the CM by ultracentrifugation according to methods described previously [14, 17]. Briefly, the CM was centrifuged at 2000 × *g* for 10 min at 4 °C. The supernatant was next passed through a 0.2-μm filter (Steradisc; Kurabo, Bio-Medical Department, Tokyo, Japan). Next, the filtrate was ultracentrifuged at 100,000 × *g* for 70 min at 4 °C (Optima XE-90 ultracentrifuge with a swing rotor, SW41Ti; Beckman Coulter, Inc., Brea, CA, USA). The precipitate was next rinsed with PBS and ultracentrifuged at 100,000 × *g* for 70 min at 4 °C. The exosome-enriched fraction was next reconstituted in PBS or D-MEM, for further studies. The protein concentration of the exosome fraction was measured using a Micro BCA Protein Assay Kit (Thermo Fisher Scientific), according to the manufacturer’s instructions. The yield of the exosome preparation was 5.8 × 10^11^–7.6 × 10^11^ particles/10^6^ cells, as determined by the electrical resistance nano pulse method (qNano; IZON Science Ltd., Oxford, UK).

CD63 is located on the limiting membranes of exosomes and MVBs; therefore, PlaMSCs were transfected with a plasmid encoding for a CD63–green fluorescent protein (GFP) fusion protein (pCT-CD63-GFP; System Biosciences, Mountain View, CA, USA) to visualize intracellular CD63 as described previously [18]. Transmission electron microscopy (TEM) was used to observe exosome morphology (Hitachi H-7100 microscope; Hitachi High-Technologies Corporation, Tokyo, Japan). The samples were prepared by dropping 4 μl of exosome solution onto a formvar-coated copper grid for 2 min at 25 °C (RT), and the samples were negatively stained with 1.5% uranyl acetate for 2 min. For immunoelectron microscopy, the samples were prepared by dropping 4 μl of exosome solution onto a formvar-coated nickel grid for 30 min at RT, and fixed in 4% paraformaldehyde in 0.1% phosphate buffer. After rinsing in 0.1 M Tris–HCl buffer, the samples were incubated with blocking solution (5% goat serum albumin) for 20 min. We next incubated the samples overnight with either anti-human CD63 antibody (1:40 dilution in 0.1 M Tris–HCl buffer; Becton, Dickinson and Company, Franklin Lakes, NJ, USA) or anti-human calnexin antibody (1:50 dilution; Proteintech Group, Inc., Rosemont, IL, USA) as positive and negative controls, respectively. After rinsing in 0.1 M Tris–HCl buffer three times, the samples were incubated with secondary antibody conjugated with 10-nm gold particles (British Bio Cell International, Cardiff, UK) for 1 h. After rinsing in 0.1 M Tris–HCl buffer, the samples were negatively stained, as already described. To evaluate particle size of exosomes, dynamic light scattering (DLS) measurements were performed using a Zetasizer Nano ZS instrument equipped with temperature control (Malvern Instruments Ltd, Malvern, UK).

### Western blot analysis

Western blotting was performed to assess for exosome marker presence. Exosomes (equivalent to 1.0 μg protein) were solubilized in sample buffer (3% sodium dodecyl sulfate, 10% glycerol, 0.05 M Tris–HCl, and 0.001% bromophenol blue) without a reducing agent for 30 min at room temperature, and separated on a 10% acrylamide gel in parallel with a molecular marker (Prestained XL-Ladder Broad, SP-2120; Apro Life Science Institute Inc., Tokushima, Japan). Proteins were then transferred to polyvinylidene difluoride membranes (Bio-Rad, Hercules, CA, USA). The membranes were blocked with 5% skim milk in Tris-buffered saline with Tween 20 overnight at 4 °C. The membranes were next incubated with a mouse anti-human CD9 IgG_1_ primary antibody solution (1:200 dilution; Santa Cruz Biotechnology, Santa Cruz, CA, USA) for 1 h at room temperature. The membranes were next incubated with a goat anti-mouse IgG horseradish peroxidase-conjugated secondary antibody (Millipore, Billerica, MA, USA) for 45 min at room temperature. Next, the membranes were incubated with the Luminata Forte substrate (Millipore), and visualized using an ImageQuant LAS 4000 mini imager (GE Healthcare, Little Chalfont, UK).

### Incorporation of exosomes

The incorporation of exosomes was examined using labeled exosomes. Exosomes were labeled with a PKH67 green fluorescent membrane linker dye (Sigma Aldrich, St. Louis, MO, USA) according to the manufacturer’s instructions. HUVECs were seeded in six-well cell culture plates (Asahi Glass Co., Ltd, Tokyo, Japan) at a cell density of 2 × 10^5^ cells/well. After a 24-h incubation, the cells were cultured in serum-free alpha-modified minimum essential media (αMEM; Thermo Fisher Scientific, Inc., Waltham, MA, USA) with the labeled exosomes (equivalent to 5.0 μg of protein) or control solution (the fluorescent membrane linker dye without exosomes). After another 24-h incubation, HUVECs were fixed in 4% paraformaldehyde (PFA) solution, and the nuclei were counterstained with Hoechst 33342. The labeled exosomes in the HUVECs were observed under a fluorescence microscope (DMI6000 B; Leica, Wetzlar, Germany) or analyzed by flow cytometry (FACSAria; BD Bioscience). The incorporation of PlaMSC exosomes (PlaMSC-exo) was independently confirmed by fluorescence microscopy or flow cytometry at least three times.

### Endothelial tube formation assay

The angiogenic activity of PlaMSC-CM or PlaMSC-exo was assessed using an in-vitro angiogenesis assay kit according to the manufacturer’s instructions (Kurabo). Either CM or exosomes was added to culture, and the endothelial cell tubes were stained with anti-human CD31 antibody and alkaline phosphatase-conjugated secondary goat anti-mouse IgG antibody after 11 days of culture. The effects of CM or exosomes on the endothelial tube formation were confirmed in the range between positive (VEGFA) and negative control (suramin). The number of endothelial cell tubes that intersected with the criteria grid (provided in the kit) was counted to quantify the effect of PlaMSC-CM or PlaMSC-exo on angiogenesis. D-MEM (*n* = 4), BMMSC-CM (*n* = 4), PlaMSC-CM (*n* = 4), and PlaMSC-exo (*n* = 4) were assaigned for the endothelial tube formation assay. All experiments were conducted independently, at least three times each.

### Cell migration assay

The migration of endothelial cells was evaluated using a scratch wound healing assay. HUVECs (8 × 10^4^ cells/well) were plated on six-well collagen type I-coated culture plates (Asahi Glass) and grown to confluence. A wound was generated by manually scratching the cell surface with a pipet tip. Subsequently, pictures of the wound area in the presence or absence of PlaMSC-exo (equivalent to 0–5.0 μg of protein) were taken under a microscope (BZ-8000; Keyence, Osaka, Japan) every 3 h for 12 h. Wound area filling by migrating cells was analyzed 12 h after treatment using the National Institutes of Health (NIH) ImageJ software at three selected points (upper, mid, and lower portions of the wound). All experiments were conducted as at least three independent replicates.

### Growth factor array

The growth factor profiles in CM prepared from BMMSCs or PlaMSCs (denoted BMMSC-CM and PlaMSC-CM, respectively) were evaluated using a multiplex growth factor array system (Human Growth Factor Antibody Array I; RayBiotech, Inc., Norcross, GA, USA). The CM was concentrated 17-fold using an ultrafilter (Amicon Ultra, 10 kDa; Millipore). The volume of the concentrated CM was calibrated according to cell numbers when the CM was collected. Colorimetric analysis using a luminescent image analyzer (ImageQuant LAS 4000 mini) was used to quantify the intensity of each membrane dot, which allowed for the assessment of growth factor content in the CM. The experiments were conducted three times using BMMSC-CM or PlaMSC-CM prepared from PlaMSCs of different patients.

### Gene expression

Fetal bovine serum (FBS) was ultracentrifuged at 200,000 × *g* for 16 h at 4 °C to deplete exosomes. HUVECs were plated in six-well culture plates at a cell density of 2 × 10^5^ cells/well in EGM2. At 70% confluence, the medium was changed to αMEM containing 5% exosome-depleted FBS, and the cells were precultured for 1 h. Next, the cells were incubated with or without exosomes (equivalent to 5.0 μg of protein) for another 72 h. In the control culture, an equivalent volume of PBS was added to the medium. Total RNA was prepared from cells using the RNeasy Mini Kit (Qiagen, Venlo, the Netherlands). Complementary DNA (cDNA) was synthesized from 1.0 μg of total RNA using a First Strand cDNA Synthesis Kit (AMV; Roche, Mannheim, Germany). The mRNA expression levels of human vascular endothelial growth factor receptor 2 (VEGFR2), human Tie-2, human angiopoietin-2 (Ang-2), and glyceraldehyde 3-phosphate dehydrogenase (GAPDH) were measured by quantitative reverse transcription-polymerase chain reaction (qRT-PCR). qRT-PCR was conducted using LightCycler FastStart DNA Master SYBR Green I reaction mix. Amplification and quantification of amplified products were performed in a LightCycler instrument (Roche). Reaction products were quantified using LightCycler Software Version 4.1 (Roche). Primer sets, annealing temperature, and references (ref) used in this study (GAPDH [14], VEGFR2, Tie2, and ANg-2 [19]) are presented in Table 1. Each experiment was independently repeated three times.

**Table 1:** Primers used for qRT-PCR

Gene	Forward primer	Reverse primer	Annealing temperature (°C)	Ref.
GAPDH	ACCACAGTCCATGCCATCAC	TCCACCACCCTGTTGCTGTA	58	15
VEGFR2	CCAAGAACTCCATGCCCCTTA	ATCCCTGGGATCTGAAACG	58	20
Tie-2	TAGAGCCTGAAACAGCATACCAGG	CTATTGGAATGGCAAATGCTGGG	61	20
Ang-2	AGCAGAAAGGATGGAGACAAC	CTTGAGCGAATAGCCTGAGC	58	20

*Ang*-*2* human angiopoietin-2, *GAPDH* glyceraldehyde 3-phosphate dehydrogenase, *qRT*-*PCR* quantitative reverse transcription-polymerase chain reaction, *ref* references, *VEGFR2* vascular endothelial growth factor receptor 2

### In-vivo angiogenesis assay

All animal study protocols and procedures were approved by the Animal Care Ethics Committee of the Tokyo Medical and Dental University (0170325A). All experiments were carried out in accordance with the approved guidelines by Science Council of Japan for proper conduct of animal experiments. The in-vivo proangiogenic activity of CM, exosome-depleted CM (CM-exo), or exosomes was evaluated using a murine auricle ischemia model. Six nude mice (8 weeks old, male) were used for the analyses in each assay. One day before exosome infusion, the proximal region on both sides of the auricular vasculature was occluded percutaneously by a 10–0 surgical suture. The CM, CM-exo, or exosomes (50 μl/day) were infused subcutaneously into the right auricles using a syringe with a 32-gauge injection needle for 2 consecutive days. PBS was injected into the auricles as a control. Superficial blood flow in the auricles was measured by laser Doppler blood flow analysis (moorLDI Laser Doppler Imager, moorLDI software version 5.1; Moor Instruments, Axminster, UK) under general anesthesia (1.5% isoflurane, 150 ml/min) before infusion (day 0), and 3 and 6 days after the second infusion. For histological analysis, the auricles were excised 3 days after the infusion of PlaMSC-exo and fixed in 4% PFA. The tissues were frozen in Tissue-Tek O.C.T. compound (Sakura Finetek USA, Inc., Torrance, CA, USA), and sectioned along the craniocaudal axis into sections 8 μm thick in a cryostat at –20 °C. The sections were stained with Mayer’s hematoxylin and eosin (HE). The histological examination was conducted under a fluorescence microscope (BZ-8000; Keyence).

### Statistical analysis

The proangiogenic activity of CM was compared to that of the control using Dunnett’s test (Fig. 2a). To assess the effect of exosome depletion of PlaMSC-CM, pairwise comparisons were made using the Tukey–Kramer adjustment for multiple comparisons (Fig. 4a). To compare the proangiogenic effect of PlaMSC-CM with that of PlaMSC-exo, Tukey–Kramer adjustments were used for multiple comparisons (Fig. 4b). The effect of PlaMSC-exo (0.2, 1.0, and 5.0 μg) compared to that of the control (0 μg) on endothelial cell migration was evaluated at 12 h using Dunnett’s test (Fig. 4c). To evaluate the effect of PlaMSC-exo on angiogenic gene expression in endothelial cells (HUVEC), Student’s *t* tests were used to compare mean values (Fig. 4e). To assess the proangiogenic effect of PlaMSC-exo in vivo, differences in blood flow values (Flux-PU) between day 0 and day 3 or 6 were evaluated. The mean Flux-PU of the PlaMSC-exo group was compared to that of the control using a paired Student’s *t* test (Fig. 5a). *P* < 0.05 was considered statistically significant.

## Results

### Phenotypic characterization of term PlaMSCs

**Fig. 1:**
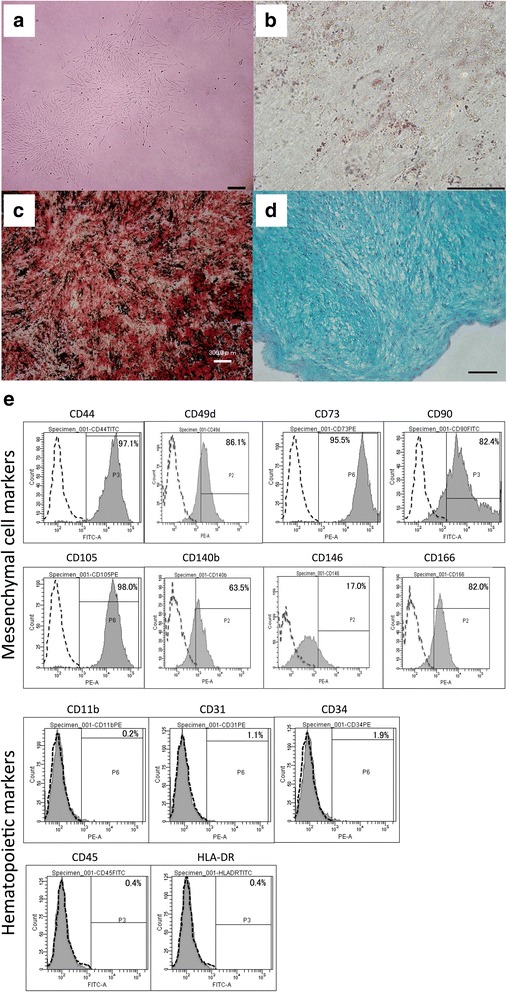
Characteristics of PlaMSCs. **a** Bright-field microscope image of colonies formed by PlaMSCs. Scale bar = 300 μm. **b** Oil Red O staining showing lipid droplet formation in PlaMSCs after adipogenic induction. Scale bar = 100 μm. **c** Calcification assessed by Alizarin Red S staining of PlaMSCs. Scale bar = 300 μm. **d** Chondrogenic differentiation of PlaMSCs assessed by Alcian blue stain. Scale bar = 100 μm. **e** Flow cytometric analysis of MSC-related cell surface markers. Dotted line histograms represent cells stained with isotype control IgGs. Solid line histograms represent cells stained with the indicated antigen-specific antibodies

Cells were isolated from human term placental tissue (chorionic plate and villus chorion) and characterized as described previously [14]. Approximately 3 × 10^7^ cells were extracted by enzymatic digestion from 10 g of minced tissue. Colonies (fibroblast colony-forming units (CFU-F)) were formed when the cells were inoculated at a density of 150 cells/cm^2^ (Fig. 1a). In addition to exhibiting colony-forming capacity, these cells exhibited adipocyte, osteoblast, or chondroblast differentiation when they were cultured in the corresponding differentiation media (Fig. 1b–d). Flow cytometric analysis was conducted to characterize the cell surface phenotype of PlaMSCs. The cells were positive for mesenchymal cell markers such as CD44, CD49d, CD73, CD90, CD105, CD140b, CD146, and CD166, and negative for hematopoietic cell markers such as CD11b, CD31, CD34, CD45, and HLA-DR (Fig. 1e). Collectively, the cells exhibited a typical MSC-like phenotype, and were designated PlaMSCs (Additional file 1: Table S1).

### PlaMSC-CM enhanced angiogenesis in vitro

**Fig. 2:**
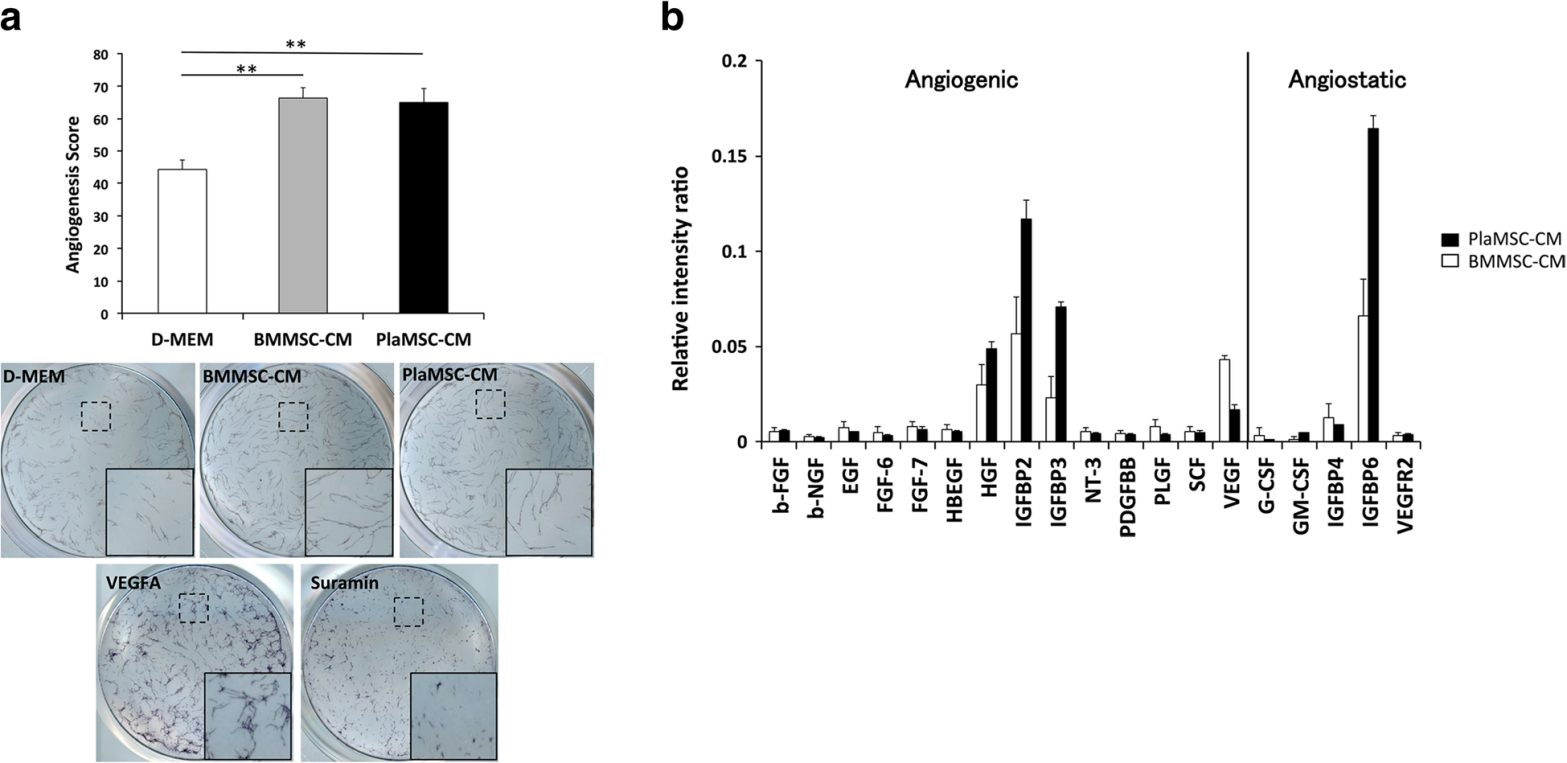
Angiogenic activity and growth factor profile of PlaMSC-CM. **a** In-vitro angiogenic activity of PlaMSC-CM assessed by an endothelial tube formation assay. Endothelial cell tubes were stained with anti-human CD31 antibody and alkaline phosphatase-conjugated secondary goat anti-mouse IgG antibody after 11 days of culture. Effects of CM on the endothelial tube formation were confirmed in the range between positive (VEGFA) and negative control (suramin). Insets show higher magnification of dotted area to demonstrate architecture of the endothelial tubular network. **P < 0.01 as determined by Dunnett’s test. **b** Growth factor array for angiogenic and angiostatic factors. Black and white bars show the relative intensity ratios of growth factors found in PlaMSC-CM and BMMSC-CM to that of the positive control, respectively. *n* = 6. Three independent experiments were performed. D-MEM Dulbecco’s modified Eagle’s medium, BMMSC-CM conditioned medium from MSCs isolated from human bone marrow, PlaMSC-CM conditioned medium from MSCs isolated from human term placental tissue, MSC mesenchymal stem cell, VEGF vascular endothelial growth factor

The effect of PlaMSC-CM on angiogenesis was assessed using an endothelial tube formation assay. VEGFA and suramin were used as positive and negative controls, respectively (Fig. 2a). It has been reported previously that BMMSCs secrete angiogenic factors such as IL-6 and VEGF [20, 21], and enhance angiogenesis in vitro [22]; thus, the proangiogenic effect of PlaMSC-CM was compared to that of BMMSC-CM. The number of endothelial cell tubes that intersected with the criteria grid was significantly increased when either PlaMSC-CM or BMMSC-CM was added to the cultures (Fig. 2a). The representative images of the tube formation assay showed that both PlaMSC-CM and BMMSC-CM increased the length and thickness of endothelial tubes compared to those in D-MEM (Fig. 2a, insets).

### BMMSC-CM and PlaMSC-CM contained angiogenesis-related growth factors

To screen the angiogenic factor(s) associated with PlaMSC-CM, a growth factor array was employed. The array of growth factors was selected based on previous reports of MSC-CM angiogenic activity using an endothelial tube formation assay. Figure 2b shows that both angiogenic and angiostatic factors were found in both BMMSC-CM and PlaMSC-CM. In BMMSC-CM, the levels of VEGF, HGF, insulin-like growth factor binding protein (IGFBP) 2 (IGFBP2), and IGFBP6 were markedly higher than those of other growth factors. In PlaMSC-CM, the levels of HGF, IGFBP2, IGFBP3, and IGFBP6 were higher than those of other growth factors.

### PlaMSC-CM showed the presence of exosomes

First, to investigate the presence of exosomes in PlaMSCs, the intracellular localization of CD63 was visualized following the transfection of PlaMSCs with pCT-CD63-GFP, as CD63 is located on the limiting membranes of MVBs and exosomes. GFP-positive signals were observed in the cytoplasm of PlaMSCs in pCT-CD63-GFP-transfected cells (Additional file 2: Figure S1), suggesting the presence of exosomes in PlaMSCs.

**Fig. 3:**
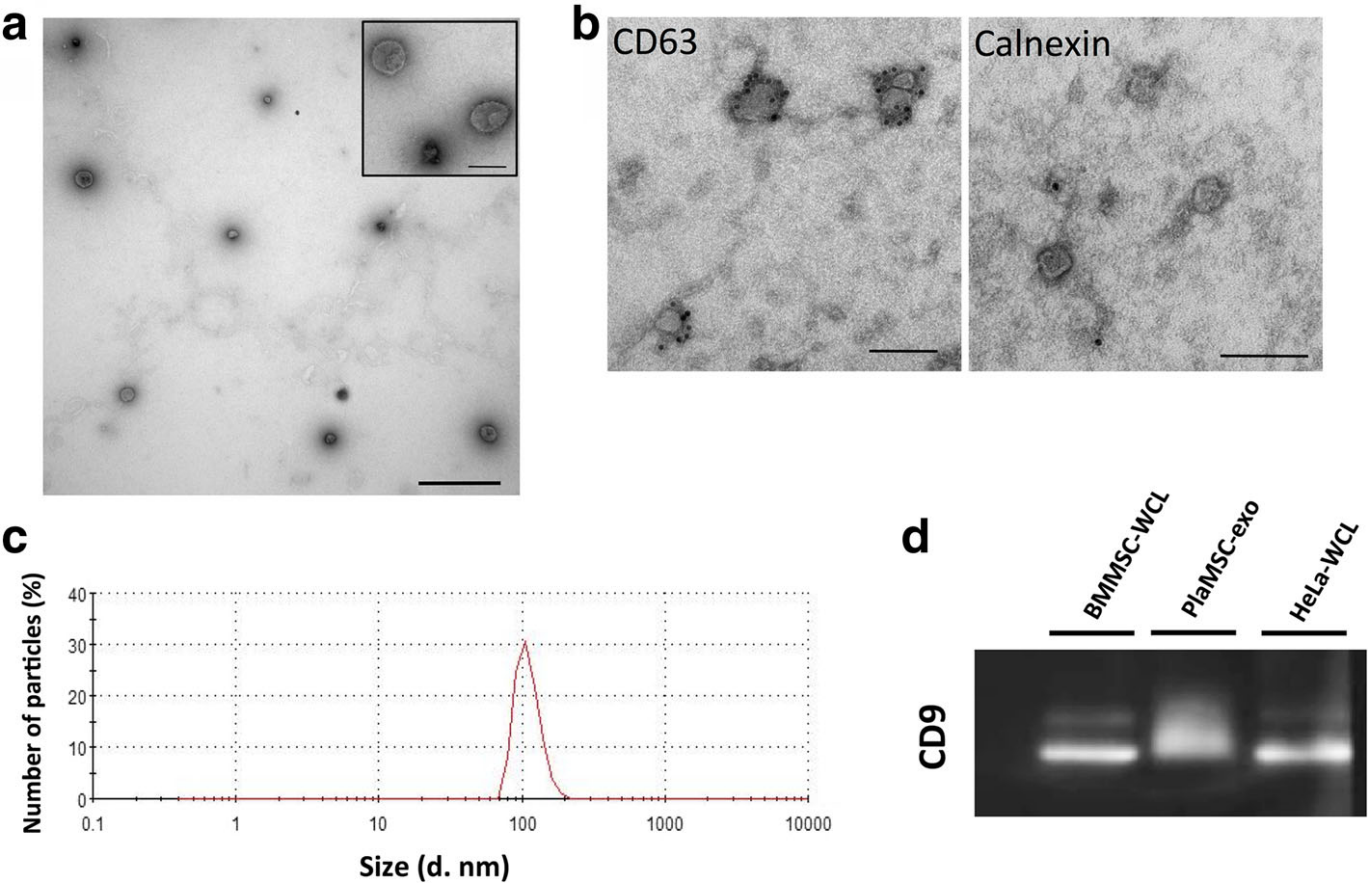
PlaMSC-derived exosomes. **a** The exosome fraction of PlaMSC-CM enriched by filtration and ultracentrifugation, and visualized by TEM with 1.5% uranyl acetate. Scale bar = 500 nm. Inset shows higher-magnification images of the exosomes, and a typical cup-shaped morphology was observed. Scale bar = 100 nm. **b** Immunoelectron microscopic images showing PlaMSC-exo are positive for CD63 but not for calnexin. CD63 used as positive control and calnexin as negative control. Secondary antibody conjugated with 10-nm gold colloidal particles was used. Scale bar = 100 nm. **c** Particle size evaluated by DLS. Data show the size of particles in the exosome fraction was approximately 100 nm in diameter. **d** Western blot analysis of the exosome marker CD9. BMMSC-WCL and HeLa-WCL used as controls. PlaMSC-exo exosomes derived from MSCs isolated from human term placental tissue, MSC mesenchymal stem cell, BMMSC human bone marrow-derived MSC, WCL whole cell lysates

Next, to examine whether PlaMSCs secrete exosomes, exosomes were recovered from the PlaMSC-CM and the morphology, size, and markers of PlaMSC-exo were identified using TEM, DLS, and western blot analyses (Fig. 3a–d). Whole cell lysates of BMMSC (BMMSC-WCL) or HeLa cells (HeLa-WCL) were used as controls. TEM of PlaMSC-exo revealed the presence of spherical structures that were ~ 50–100 nm in diameter (Fig. 3a). The inset in Fig. 3a shows the cup-shaped morphology of the vesicles, which is typical of exosomes prepared by ultracentrifugation. The vesicles were analyzed by immunoelectron microscopy with anti-CD63 or anti-calnexin antibody as positive and negative controls, respectively. This microscopy demonstrated that the vesicles were immunogold-labeled with anti-CD63 antibody, but not with anti-calnexin antibody (Fig. 3b). DLS measurements verified the vesicle size distribution (Fig. 3c). The western blot analysis revealed the presence of CD9, a widely accepted exosome marker, in PlaMSC-exo under nonreducing conditions (Fig. 3d and Additional file 3: Figure S2).

### PlaMSC-exo demonstrated a proangiogenic effect

**Fig. 4:**
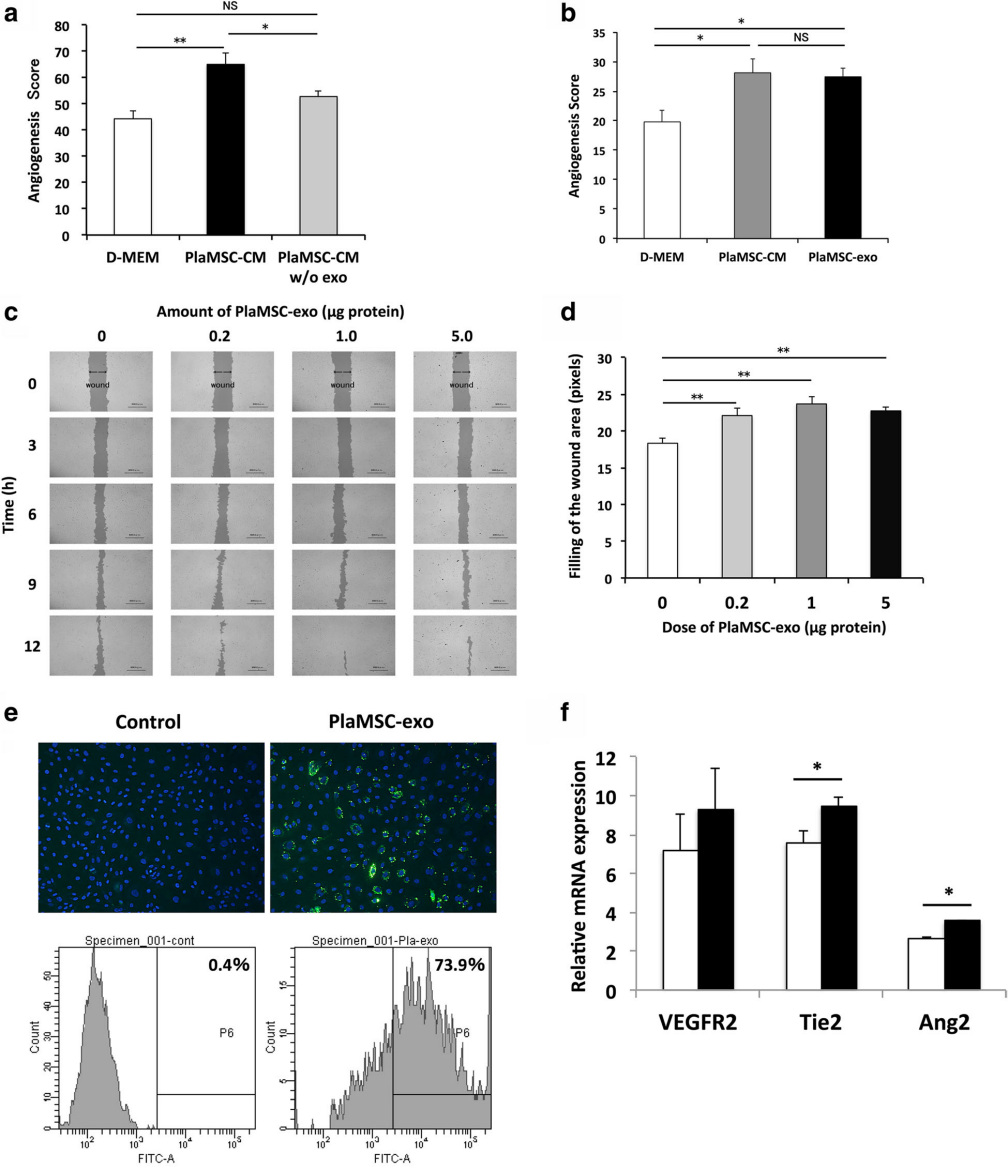
Angiogenic activity of PlaMSC-exo. **a** Endothelial tube formation assay revealed reduced angiogenic activity of PlaMSC-CM after depletion of the exosome fraction. White, black, and gray bars show D-MEM, PlaMSC-CM, and PlaMSC-CM without exosomes (w/o exo), respectively. **b** PlaMSC-exo enhanced angiogenesis with comparable efficiency to that of PlaMSC-CM. White, gray, and black bars shows D-MEM, PlaMSC-CM, and PlaMSC-exo, respectively. **c** Migration of endothelial cells enhanced by PlaMSC-exo. Endothelial cell migration was evaluated using a scratch wound healing assay. Pictures of the wound area in the presence or absence of PlaMSC-exo (equivalent to 0, 0.2, 1.0, or 5.0 μg of protein) taken under a microscope every 3 h for 12 h. Representative data from three independent experiments. **d** Scratch wound healing assay showing PlaMSC-exo enhanced endothelial cell migration. Wound area filling by migrating cells was quantitated 12 h after treatment at three selected points (upper, mid, and lower portion of the wound). Quantitative data from the scratch wound healing assay shown in pixels. Data expressed as mean ± SE (0 μg, *n* = 4; 0.2 μg, 1.0 μg, and 5.0 μg, *n* = 5 in each experiment). *P < 0.05, **P < 0.01, determined by Dunnett’s tests. NS not significant. **e** Fluorescent microscope image showing incorporation of PKH67-labeled PlaMSC-exo (upper panel). HUVECs were exposed to PlaMSC-exo or control solution for 24 h and fixed, which was subjected to flow cytometric analysis of the incorporation of PlaMSC-exo by endothelial cells (lower panel). Mean frequency of fluorescent signals indicated. **f** PlaMSC-exo enhance expression of angiogenesis-related genes in HUVECs. HUVECs were exposed to PlaMSC-exo (black bar) or PBS (white bar) for 72 h, and then harvested for isolation of total RNA, which was subjected to qRT-PCR analyses of angiogenesis-related gene expression. Each experiment independently repeated three times. Expression level of each mRNA normalized to that of GAPDH mRNA. Data expressed as mean ± SE. *P < 0.05, determined by Student’s *t* tests. D-MEM Dulbecco’s modified Eagle’s medium, PlaMSC-CM conditioned medium from MSCs isolated from human term placental tissue, MSC mesenchymal stem cell, PlaMSC-exo exosomes derived from MSCs isolated from human term placental tissue, Ang-2 human angiopoietin-2, VEGFR2 human vascular endothelial growth factor receptor 2

As shown in Fig. 2a, PlaMSC-CM enhanced angiogenesis in vitro. The increased proangiogenic effect of PlaMSC-CM compared to that of the control (D-MEM) was reduced by approximately 60% when exosomes were depleted from the CM by ultracentrifugation (Fig. 4a). Moreover, PlaMSC-exo (1.0 μg) enhanced angiogenesis in vitro to the same extent as PlaMSC-CM (Fig. 4b). Collectively, these results suggest that the proangiogenic effect of PlaMSC-CM was partly due to the exosomes.

Furthermore, the scratch wound healing assay demonstrated that the exosomes (0.2–5.0 μg) significantly enhanced the migration of endothelial cells compared to that of the control (Fig. 4c, d). Both fluorescence microscopy and flow cytometry demonstrated the incorporation of labeled PlaMSC-exo into endothelial cells after 24 h of incubation (Fig. 4e). qRT-PCR analysis showed that there was a significant increase in expression of the angiogenic markers Tie2 and Ang2 in endothelial cells treated with PlaMSC-exo (Fig. 4f). Collectively, these results suggest that the incorporation of PlaMSC-exo enhanced the angiogenic activity of endothelial cells in vitro.

### PlaMSC-exo demonstrated in-vivo angiogenic activity

**Fig. 5:**
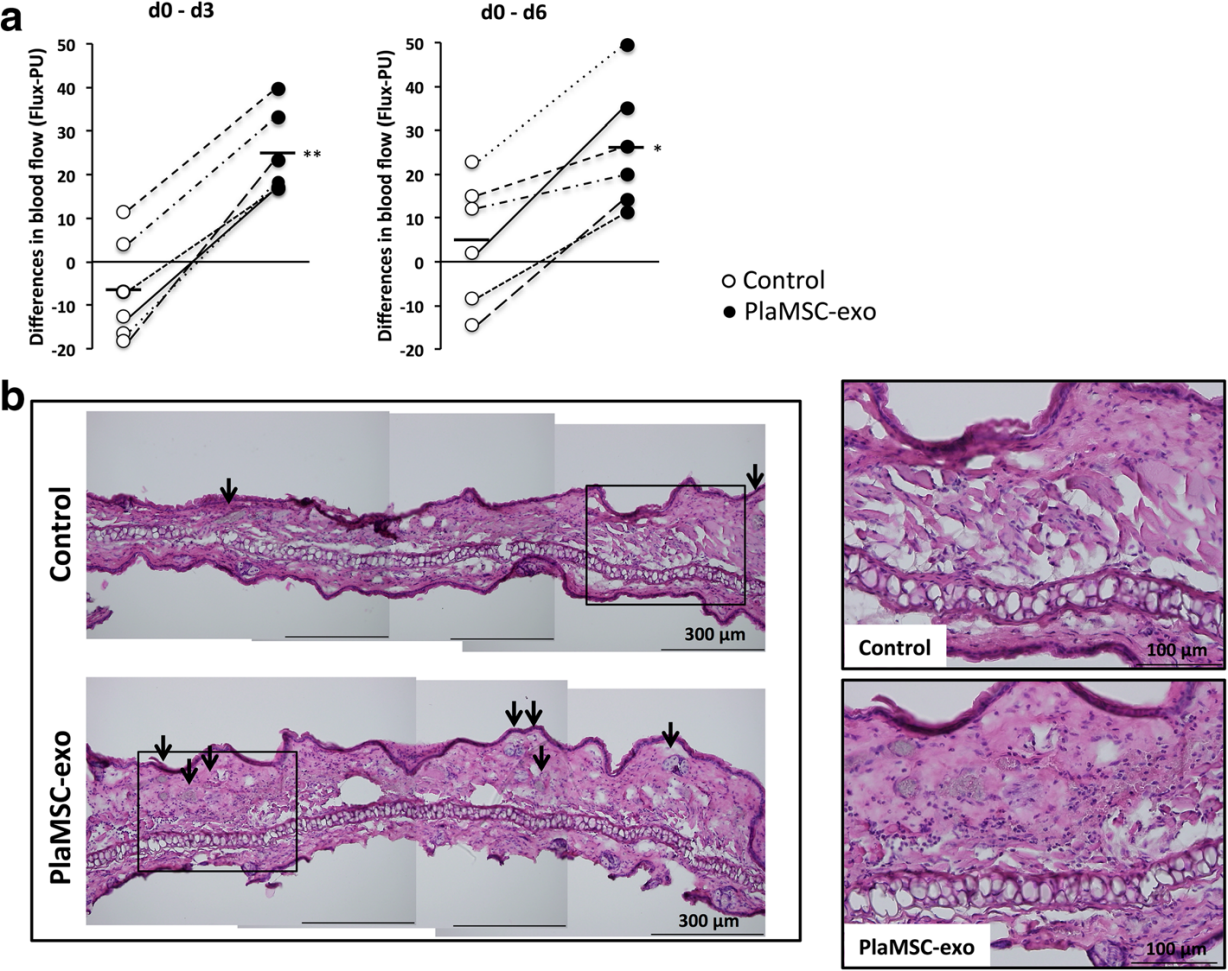
Angiogenic activity of PlaMSC-exo in vivo. **a** Laser Doppler blood flow analysis. Superficial blood flow on auricles significantly increased on days 3 and 6 following infusion of PlaMSC-exo (4.4 μg total protein, closed circles) compared to control (open circles). Six mice were used for the analyses. Mean differences in blood flow (Flux-PU) between day 0 and day 3 or 6 were compared between PlaMSC-exo and controls. A line with the same style denotes the same animal. Means indicated by horizontal lines. *P < 0.05, **P < 0.01, determined by Student’s *t* tests. **b** Representative histological sections of murine auricles. Murine auricles were excised 3 days after infusion of PlaMSC-exo and the frozen sections were stained with HE for histological examination. Infusion of PlaMSC-exo (4.4 μg in total) resulted in increased small blood vessels as indicated by the arrows. PBS (–) was infused as negative control. Scale bar = 300 μm. Right panels show enlarged images of square regions in left panels. Scale bar = 100 μm. PlaMSC-exo exosomes derived from MSCs isolated from human term placental tissue

The angiogenic activity of PlaMSC-CM, exosome-depleted PlaMSC-CM, or PlaMSC-exo was analyzed using an in-vivo murine auricular ischemic injury model. PlaMSC-CM, exosome-depleted PlaMSC-CM, or PlaMSC-exo was injected subcutaneously into auricular wounds 1 day after vessel occlusion for 2 consecutive days. Blood flow was monitored by laser Doppler (Flux-PU, mean ± standard error). In controls, some mice exhibited slight recovery in blood flow. Other control mice showed a continuous decrease in blood flow (Fig. 5a open circles). The increased blood flow by PlaMSC-CM was significantly reduced when exosomes were depleted from the CM at day 3 (32.53 ± 6.85 vs 2.87 ± 7.52) and day 6 (36.43 ± 6.91 vs 7.68 ± 7.02) (*n* = 6, *P* < 0.05). Moreover, PlaMSC-exo significantly increased peripheral blood circulation at both 3 and 6 days after injection (Fig. 5a closed circles) compared to that of controls (Fig. 5a open circles). In HE-stained histological sections, the number of small blood vessel-like structures was increased following subcutaneous infusion of PlaMSC-exo into the murine auricles (control (*n* = 2) 8.0 ± 2.8 vs PlaMSC-exo (*n* = 3) 13.6 ± 2.5) (Fig. 5b).

## Discussion

Several previous studies have demonstrated enhanced angiogenesis due to term PlaMSCs and their CM. However, the mechanisms underlying the proangiogenic effects of PlaMSCs or PlaMSC-CM have remained elusive.

The results of the current study indicate that PlaMSC-exo are partly responsible for the angiogenic effects of PlaMSC-CM in vitro and that PlaMSC-exo also enhance angiogenesis in vivo. As shown in Fig. 3a, microvesicles recovered from the CM were heterogeneous in size (approximately 50–100 nm in diameter). Immunoelectron microscopic images of PlaMSC-exo show that the microvesicles were positive for CD63 but not for calnexin (Fig. 3b), demonstrating that exosomes may be a component of the collected fraction. Furthermore, we found that the depletion of the exosomes significantly reduced the proangiogenic effects of PlaMSC-CM. Preliminary data showed that the expression profile of cytokines and growth factors in PlaMSC-CM was not markedly changed after depletion of exosomes (data not shown). Additionally, PlaMSC-exo were incorporated into endothelial cells, where they induced migration, tube formation, and angiogenic gene expression (Fig. 4a–f), suggesting that the angiogenic effects of PlaMSC-CM were partly due to the direct stimulation of endothelial cells by exosomes.

Although we find that both PlaMSC-CM and BMMSC-CM enhanced endothelial cell tube formation, the secreted growth factor profiles between these two media were markedly different. For example, VEGF was predominant in BMMSC-CM, whereas IGFBP2 was the major proangiogenic growth factor in PlaMSC-CM. Our preliminary data showed that the tube formation stimulated by PlaMSC-CM was slightly inhibited in the presence of a neutralizing antibody against VEGF, although this inhibition was not significant (data not shown). Taken together, these data imply that the enhanced tube formation by the CM was due to the effects of both proangiogenic and angiostatic factors in the CM, or novel mechanisms such as exosomes.

Recently, our group reported that PlaMSC-exo altered the competence of fibroblasts to differentiation stimuli [14]. PlaMSC-exo upregulated the transcriptional activity and mRNA expression of the stemness-related gene, *OCT4*, in fibroblasts; thus, PlaMSC-exo may also regulate the responsiveness of endothelial cells to proangiogenic growth factors. Examining the effects of PlaMSC-exo on the responsiveness of endothelial cells to angiogenic growth factors in CM would therefore be informative. Our data herein also indicate that injection of PlaMSC-exo significantly enhanced angiogenesis in the murine auricle wound model. Auricle vessels were occluded 1 day prior to the injection to cause ischemic injury and subsequent angiogenesis. Under these conditions, PlaMSC-exo stimulated angiogenesis. Vasodilation was also observed when PlaMSC-exo were injected (data not shown). You et al. [23] reported that administration of BMMSCs promoted vasodilation via the release of nitric oxide (NO) in a murine hind-limb ischemia model, suggesting that the proangiogenic activity of MSCs may rely on their capacity to modulate the function of preexisting vessels. It would thus be interesting to determine, in our auricular wound model, whether PlaMSC-exo induces endothelial NO synthase (eNOS) to release NO.

The similarities and/or disparities between BMMSCs and PlaMSCs have been reported previously [24–26]. Shabbir et al. [27] reported that the uptake of BMMSC-exo by HUVECs resulted in an increase in tube formation. Lai et al. [12] reported that exosomes secreted from embryonic stem cell-derived MSCs reduced the infarct size in a murine myocardial ischemia/reperfusion injury model. Salomon et al. [28] reported that exosomes of placental villi-derived MSCs enhanced migration and tube formation of endothelial cells in vitro, and that the number of exosomes released from the cells increased under hypoxic conditions. Exosomes contain various molecules such as proteins, mRNA, and miR, and may exert their biological effects on cells by transporting these molecules [29, 30]. However, the mechanisms by which PlaMSC-exo enhance the angiogenic activity of endothelial cells are under continued study. Squadrito et al. [31] have reported that parent cells have a regulatory mechanism for allowing specific intracellular miR to enter exosomes. Therefore, it would be interesting to compare proportions of miR among MSCs derived from various tissues, to find common or cell-specific miR with proangiogenic activity.

One limitation of this study is that we used a basic centrifugation protocol [17] to recover exosomes from PlaMSC-CM, which may have permitted contamination by other nonexosome vesicles and/or macromolecular aggregate in the exosome fraction. Recent studies have shown that the purity of exosomes was improved by adding to the basic centrifugation protocol a purification step using a 30% sucrose/distilled H_2_O cushion. Therefore, further studies are needed to improve the purity of PlaMSC-exo and to elucidate the proangiogenic factors of PlaMSC-exo.

The mechanisms underlying PlaMSC-exo-stimulated angiogenic activity in endothelial cells remain unclear, and further examination is required. Nevertheless, the findings of the present study indicate that PlaMSC-exo stimulated angiogenesis in vitro and in vivo. Our findings suggest that the application of PlaMSC-exo is a promising alternative treatment for ischemic disease.

## Conclusions

The present study demonstrated that PlaMSC-CM contains extracellular vesicles including exosomes, and that PlaMSC-exo exert proangiogenic effects on endothelial cells. In addition, PlaMSC-exo enhanced angiogenesis in a murine auricle ischemia model, suggesting that these exosomes may be involved in the proangiogenic activity of PlaMSC-CM. Instead of the application of a single angiogenic growth factor, PlaMSC-exo may be an alternative treatment for ischemic disease.

## Additional files

Additional file 1: Table S1. Characteristic of PlaMSCs. (TIFF 103000 kb)

Additional file 2: Figure S1. Intracellular localization of CD63. (TIFF 103000 kb)

Additional file 3: Figure S2. Western blot analysis of exosome marker CD9. (TIFF 103000 kb)

## Abbreviations

Ang-2: Human angiopoietin-2
bFGF: Basic fibroblast growth factor
BMMSC: Human bone marrow-derived MSC
CD: Cluster of differentiation
cDNA: Complementary DNA
CFU-F: Fibroblast colony-forming units
CM: Conditioned medium
DLS: Dynamic light scattering
D-MEM: Dulbecco’s modified Eagle’s medium
eNOS: Endothelial nitric oxide synthase
FBS: Fetal bovine serum
GAPDH: Glyceraldehyde 3-phosphate dehydrogenase
GFP: Green fluorescent protein
HE: Hematoxylin and eosin
HGF: Hepatocyte growth factor
HUVEC: Human umbilical vein endothelial cell
IGF-1: Insulin-like growth factor-1
IGFBP: Insulin-like growth factor binding protein
IL: Interleukin
MCP-1: Monocyte chemoattractant protein 1
miR: MicroRNA
MSC: Mesenchymal stem cell
MVB: Multivesicular body
NIH: National Institutes of Health
NO: Nitric oxide
PBS: Phosphate-buffered saline
PFA: Paraformaldehyde
PlaMSC: MSC isolated from human term placental tissue
PlaMSC-CM: Conditioned medium from PlaMSCs
PlaMSC-exo: PlaMSC-derived exosomes
qRT-PCR: quantitative reverse transcription-polymerase chain reaction
ref: Reference
RT: Room temperature
TEM: Transmission electron microscopy
TGF-β: Transforming growth factor beta
VEGF: Vascular endothelial growth factor
VEGFR2: Human vascular endothelial growth factor receptor 2
WCL: Whole cell lysates
